# Is There Successful Aging for Nonagenarians? The Vitality 90+ Study

**DOI:** 10.1155/2012/868797

**Published:** 2012-10-16

**Authors:** Lily Nosraty, Tytti Sarkeala, Antti Hervonen, Marja Jylhä

**Affiliations:** ^1^Gerontology Research Center and School of Health Sciences, 33014 University of Tampere, Tampere, Finland; ^2^Finnish Cancer Registry, Institute of Statical and Epidemiological Cancer Research, Pieni Roobertinkatu 9, 00130 Helsinki, Finland

## Abstract

*Objectives*. This study was designed (1) to estimate the prevalence of successful aging among nonagenarians based on six different models and (2) to investigate whether successful aging is associated with socio-demographic factors. *Methods*. A mailed survey was conducted with people aged 90+ in Tampere in 2010. Responses were received from 1283 people. The prevalence of successful aging was measured by six multidimensional models including physical, social, and psychological components. Age, sex, marital status, level of education, and place of living were studied as factors associated with successful aging. *Results*. The prevalence of successful aging varied from 1.6% to 18.3% depending on the model applied. Successful aging was more prevalent in men, and also more prevalent among community-living people. In most models, successful aging was also associated with younger age, being married, and a higher level of education. *Discussion*. Models which emphasize the absence of disease and activity as criteria for successful aging may not be the most relevant and applicable in oldest old. Instead, preference should be given to models that focus more on autonomy, adaptation and sense of purpose. Age-sensitive approaches would help us better understand the potential of successful aging among individuals who already have success in longevity.

## 1. Introduction

Increasing longevity is one of the great achievements of our civilization, but it has also given rise to discussion about good and successful aging. The concept of successful aging has attracted much debate, but there is still no universally accepted definition or standard measurement tool for it. The Encyclopedia of Aging defines successful aging as survival (longevity), health (lack of disabilities), and life satisfaction (happiness) [1]. It appears that the main sources of difficulty lay in the ambiguity of the meaning of “success,” in the complexity of the aging process, the rapid changes taking place in society, and the changing characteristics of the older population.

Discussions on successful aging have taken two main perspectives: one defines successful aging as a state of being, while the other understands it as a process of adaptation, described as doing the best with what one has [2]. Studies taking the adaptation approach have often found that older people themselves feel they are aging successfully, even though traditional quantitative models say otherwise [3, 4]. Successful aging as a state of being, then, is an objective measurable condition at a certain point in time, demonstrating the positive extreme of normal aging. The most influential model of successful aging as a state of being was introduced by Rowe and Kahn [5–8], who characterize “success” as absence of disease and disability, maintained physical and mental functioning, and active engagement with life. Many studies and definitions take the view that successful aging is possible only among individuals without disease and impairment. Obviously such categorizations are likely to exclude most older people, typically the oldest-old, from the possibility of successful aging [9].

Successful aging is of course impossible in the absence of aging. Still, according to Bowling [3], longevity is only rarely mentioned in lay or biomedical definitions. In studies using quantitative measures, younger age is one of the most regular predictors of successful aging [10, 11], and the rate of “success” drops dramatically in very old age. This may largely be due to the usual focus on physical deficits. Indeed, several researchers have emphasized the need to use multidimensional models and to adopt different conceptual approaches to studying different age groups [3, 12]. Recently, Young et al. [13] suggested that successful aging may coexist with diseases and functional limitations if compensatory psychological and social mechanisms are used. Their model considers three important principles: the heterogeneity of aging, multiple pathways to successful aging, and individual compensation mechanisms to adjust for age-related changes.

The oldest-old group of nonagenarians meets the key biomedical criterion of successful aging that is longevity. They are also a rapidly growing age group that is heterogeneous in terms of health and functioning: a large majority have some health problems but are independent in basic everyday activities [14].

In this study, we investigate successful aging in an unselected population of nonagenarians, applying several different models that include physical, social, and psychological dimensions. The models differ with respect to the threshold for “success” on the physical, social, and psychological dimensions. Our aim is not to introduce an ideal or universal model, but rather to demonstrate the variation in the prevalence of successful aging by applying different criteria. The first objective of this study was to construct six different models of successful aging and to use these models to estimate the prevalence of successful aging among nonagenarians. The second objective was to investigate whether successful aging in nonagenarians, defined in several different ways, is associated with sociodemographic factors.

## 2. Methods

### 2.1. Data

The Vitality 90+ study is a population-based multidisciplinary research program on nonagenarians in the city of Tampere, Finland. In the context of this program, mailed surveys were conducted with all community-dwelling people in 1996 and 1998, and with both community-dwelling and institutionalized people four times since 2001. This study used the data from the mailed survey in 2010. A questionnaire was sent to all individuals aged 90 or over in Tampere (*N* = 1630). Responses were received from 1283 people, giving a response rate of 79%. Proxy responses were obtained from 22% of the subjects who were themselves unable to answer the questions. For additional 20%, the respondent chose the answers but someone else helped in reading the questions or writing down the answers.

The research protocol was approved by the City of Tampere Ethics Committee. Informed consent was obtained from all respondents or their legal representatives.

### 2.2. Independent Variables

We explored the associations of five sociodemographic factors with successful aging: age, sex, marital status, level of education, and place of living. Age was categorized into three groups: 90-91, 92-93, and 94–107. Marital status was classified as currently married and currently unmarried, including never married, divorced, and widowed. Education was categorized into four groups as low (no more than elementary schooling), middle (lower secondary school), high (vocational school, folk high school, or upper secondary school), and highest (college and academic education). Place of living was dichotomized as community (private and service housing) and institution (residential care, service housing with 24-hour assistance, and hospitals).

### 2.3. Components of Successful Aging

Our dependent variable was successful aging. It was described by six different models that were constructed using psychical, social, and psychological indicators.

The physical component included three elements: diseases, functioning, and senses. The participants were asked whether they had been told by a doctor that they had (1) a heart problem, (2) stroke, (3) circulatory problems in the brain, (4) diabetes, (5) arthritis, (6) Parkinson's diseases, (7) hip fracture, or (8) dementia or memory problems. For the measurement of functional ability, the participants were asked whether they were able to perform independently (a) three mobility activities: moving about indoors, walking 400 meters, using stairs and (b) two ADL activities: getting in and out of bed and dressing and undressing. The response options, (1) yes, without difficulty; (2) yes, with difficulty; (3) only with help; (4) not at all, were categorized as independent (1 + 2) and dependent (3 + 4). The participants were also asked whether they were able to read the newspaper, with glasses if they used glasses (vision), and to hear what another person was saying when they were alone with them, with hearing aid if they used a hearing aid (hearing).

The psychological component was described by three variables. The participants were asked whether they suffered from depression or had depressive feelings (yes, no). Present self-rated health was categorized as average or good (very good, fairly good, and average) and poor (fairly poor and poor). Self-rated health was included in the psychological components because it is a subjective measure with no predetermined criteria: it reflects not only the more objective components of health, but also and importantly the age-related way in which the individual adjusts and adapts to different health problems [15]. The participants were also asked whether they thought it was good for people to live to be 100 years (yes, no).

The social component was measured by two questions: the frequency of meetings with children (six categories from today or yesterday to several years ago) and the frequency of talking on the phone with family members or friends (six categories from today or yesterday to several years ago). One-fifth (20.1%) of the respondents had no children. If these participants had had telephone contacts during the past two weeks, they were categorized as having had contact with children.

The percentage of missing data varied between the different variables. The highest figures were recorded for two psychological variables. Part of the reason for this was that these questions were not asked of proxy respondents. Most of these participants lived in institutions and had multiple health problems. To avoid reducing the number of participants in the analyses, we categorized both proxy responses and other missing values in these two variables at the negative extreme (poor self-rated health and thinking that it is not good to live to be 100). This imputation was done to avoid overestimation of the prevalence of successful aging, which would happen if the frailest participants were lost from the analyses.

### 2.4. Constructing Six Models of Successful Aging

**Figure 1:**
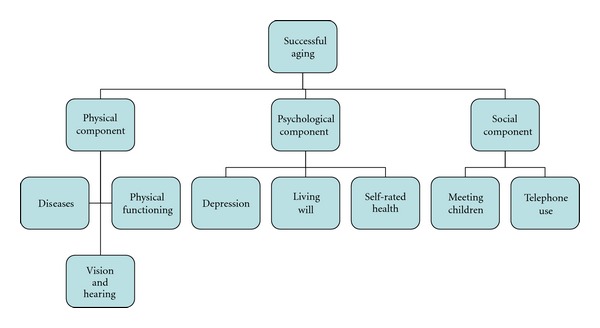
Three components of successful aging.

Following Rowe and Kahn [6] and Young et al. [13], we defined successful aging as consisting of three components as shown in Figure 1. Six different models were constructed with different thresholds. The main differences between the models are in the physical component, where we defined four alternative criteria for “success,” ranging from most to least demanding as follows:

 Criterion 1: absence of disease + good vision and hearing + independence in all five activities.
 Criterion 2: less than three diseases, no dementia, good vision and hearing, and independence in ADL and moving about indoors (independent in 3 easier activities).
 Criterion 3: no dementia, good vision and hearing, and independence in all five activities.
 Criterion 4: good vision and hearing, and independence in all five activities.

In the psychological component, “success” was defined as absence of depressiveness, average or good self-rated health, and agreement with the view that it is good to live to be 100. In the social component, “success” was defined as having met one's children and having talked on the phone with family members or friends during the past two weeks.

The six models of successful aging were constructed as follows:

 Model 1: Physical component criterion 1 and psychological component & social component.
 Model 2: Physical component criterion 2 and psychological component & social component.
 Model 3: Physical component criterion 3 and psychological component & social component.
 Model 4: Physical component criterion 4 and psychological component & social component.
 Model 5: Physical component criterion 3 and psychological component.
 Model 6: Physical component criterion 3 and social component.

### 2.5. Analysis

The prevalence of successful aging in different sociodemographic categories was compared by cross tabulation using the Chi-square test. Logistic regression models were used to assess the independent associations of different models of successful aging with sociodemographic factors. Odds ratios (ORs) and 95% confidence intervals (95% CI) were calculated. These analyses were performed using the SPSS Package 16.

## 3. Results

Most of the participants (85.9%) were under 95 years of age, and more than 80% were women. These figures well reflect the distributions in the general population. Only 12.1% were still married and 37.5% lived in an institution. The majority had no more than elementary schooling (Table 1). Heart problems, arthritis, and dementia were the most frequent diseases, and only 14.7% of men and 10.2% of women did not have any of the eight conditions listed in the questionnaire. Four in ten respondents were independent in all five activities, and seven in ten were independent in ADL and moving about indoors. According to different criteria, 5.3 to 25.2% were aging successfully if only the physical component was considered. In the psychological component, the prevalence of successful aging was 20%, in the social component the figure was markedly higher at 75%. Men had better scores than women in both the physical component (most criteria) and the psychological component (Table 2).

The prevalence of successful aging varied between the six models (Table 3). It was lowest (1.6%) for Model 1, which required absence of all diseases, independence in all five activities, and good vision and hearing, in addition to the psychological and social components, and highest (18.3%) for Model 6, which differed from Model 1 in that diseases other than dementia were allowed, and the psychological component was not included. Successful aging was significantly more prevalent in men than women and among community-living than institutionalized individuals, regardless of the model. According to most models, successful aging was more frequent among those aged 90–93 than those aged 94+, among married people, and among those with a higher education.

Finally, logistic regression models were calculated to examine the independent association of different sociodemographic indicators with the six models of successful aging (Table 4).

In four models, higher age was independently associated with less successful aging. Gender was another predictive variable, and in all models except model 6, men were significantly more successful in aging than women. Higher education was a significant predictor in two models, and in Model 6 both those with a high and the highest educational level differed significantly from those with the lowest level of education. Marital status did not play an independent role, but place of living was a significant determinant of successful aging in all but Model 1.

## 4. Discussion

This paper examined one the most prominent concepts in aging research, successful aging, by constructing six different models to measure it among nonagenarians. The models were based on work by Rowe and Kahn, Rowe, and Young et al. [6–8, 13, 16], although not the exact same indicators were used. According to Young et al. [13] and Rowe and Khan [6], successful aging is typically understood as comprising three main domains: physical (in Young et al.: physiological), psychological, and social (in Young et al.: sociological). The results showed that the prevalence of successful aging varies markedly from one model to another, standing at 1.6% for Model 1 that required the absence of any disease, independence in functioning, and the ability to hear and read, as well as meeting the psychological and social criteria, and at 18.3% for Model 6, which required the absence of dementia, independence in functioning, the ability to hear and read, and meeting the social criteria. However, the main socioeconomic predictors remained largely the same across the models.

It is obvious that the absence of disease is the most demanding criterion for measuring successful aging. Disease and at least some functional deterioration are almost inevitable in very old age. Only 11% of the nonagenarians in our study had no major disease, and only 5.3% were both free of disease, able to hear and see, and independent in five daily activities (physical dimension criterion 1). Very few earlier studies have attempted to estimate the prevalence of successful aging in nonagenarians or in very old age in general. von Faber et al. [2] classified only 10% of community-dwelling and 1.9% of institutionalized participants aged 85 or over as successful agers. In the NonaSantfeliu study by Formiga et al. [17], the figure was 12% with community-dwelling nonagenarians. These studies emphasized the role of health and physical functioning, but also included some social or quality-of-life measures. It is clear that especially when the focus is on the physical dimension, successful aging will be very rare among people experiencing longevity.

Rowe and Kahn [6] included productive activities in their model of successful aging but these can hardly be expected from nonagenarians. Horgas et al. [18] showed that the daily activities of individuals aged 90 or over differed from other age groups, and in all categories this age group was engaged in significantly less activity than others. This implies that the social dimension of successful aging among the oldest old should be measured using different criteria and against different activities than in the case of the younger old and should be seen in relation to the situation of the best performers in the same age group.

In cross-sectional analysis, we limited our examination to socioeconomic predictors that at least potentially have played a role in the lives of the individuals for a longer time, and, with the exception of place of living, are not supposed to be influenced by factors that were thought to be components of successful aging. In most studies age has emerged as one of the strongest predictors of successful aging [18]. In our study, persons aged 94 or over were less likely to meet the successful aging criteria than the younger age groups. The difference between the age groups was significant for all except Model 1, and it was greatest in Model 6 where the overall prevalence of successful aging was highest. After adjusting for other sociodemographic variables, a significant age difference still persisted in four models.

In our study, the prevalence of successful aging was consistently higher for men, and in all except the last model the differences were also significant after the adjustments. Earlier studies [10] show no consistent patterns of gender differences, but the results seem to be dependent on the model used. McLaughlin et al. [11] found no gender difference in prevalence, but higher odds of successful aging in women after controlling for sociodemographic variables. Our findings among nonagenarians are only partly explained by the high prevalence of disabilities and disease in women, as men had clearly better scores in the psychological component as well. These disparities are likely to reflect differential survival, lifelong differences in biological, health, and social conditions.

Marital status was associated with successful aging in unadjusted analysis but not in the adjusted models, where the uneven age and gender distribution of the variable was controlled for.

Education is known to have an impact on health and life style, and it reflects socioeconomic status; therefore, it can also be considered a potential predictor of successful aging. Most of the studies reviewed by Depp and Jeste [10] found no differences according to educational level, but the analysis by McLaughlin et al. [11] in the Health and Retirement Study showed that the prevalence of successful aging was higher in groups with a higher education and household income. The study of Pruchno et al. [19] revealed that a higher level of formal education is associated with successful aging. Our findings with an older group than in these studies showed a graded increase in the prevalence of successful aging with higher education, although the difference was not significant for all models. The discrepancy between the findings may at least partly be due to sampling bias. In several studies institutionalized people and those of lower social position were less likely to participate [10], while our study represents the whole age group in the region.

Place of living is not usually considered a predictor of successful aging and in many (but not all, see e.g., von Faber et al. [2]) studies samples only include community-dwelling individuals. In our study, we wanted to take account of the possibility of successful aging even in an institution. However, the results showed that the prevalence of successful aging was clearly lower for those living in institutions, and this was also true for the adjusted models. Our earlier analyses (not shown here) indicated that disease, disability, and problems with hearing and seeing are more prevalent in institutions, as is self-rated health, which partly explains this finding.

### 4.1. Strengths and Limitations

The major strength of this study is that it covers the whole population aged 90 or over in the area concerned, including institutionalized people as well as proxy responses. The response rate was high. Our earlier and ongoing analyses suggest that the information on health and functioning collected by mailed questionnaires among nonagenarians is sufficiently valid and reliable [20, 21]; particularly as for a majority for those suffering from dementia, the answers were given by a proxy respondent.

In order to gain a broad and thorough understanding of successful aging, we included both physical, psychological, and social components in our analyses. Unlike most other studies, we also included the ability to see and hear as an important contributing factor to independence and quality of life. The main limitations of our study have to do with the measures used to assess the social and psychological components. Our only information about meeting with other people concerned meetings with children; no data were available about other family members or friends. One fifth of the respondents had no children, and we decided to give them a positive score for social contacts if they had made or received any telephone calls during the past two weeks. One-fifth of our responses were from proxies, who were not asked about self-rated health or living to be 100. Therefore, we had a high percentage of missing or proxy answers to two questions regarding the psychological dimension of successful aging. In order not to overestimate the prevalence of successful aging, we scored this missing data and proxy answers as negative. These kinds of problems are unavoidable in unselected samples of very old people, but they nonetheless add some uncertainty to our findings. Another obvious limitation of our study is that we had no direct questions designed to capture our respondents' self-evaluations of their life.

### 4.2. Implications

Our study in a nonselected population of persons aged 90 or over supports earlier findings that the prevalence of successful aging is highly dependent on the model applied, but in every case successful aging is associated with age, gender, and socioeconomic status. However, it is apparent that with any model that defines successful aging as a state of being and that uses criteria commonly used for younger age groups, successful aging remains a rare situation among the oldest old. An increased likelihood of health and functional problems, often followed by reduced opportunities for active social engagement, is normative consequences of biological aging and typical of extreme longevity. Therefore, in very old age, rather than models emphasizing the absence of disease and activity, emphasis should be given to approaches focusing on autonomy, adaptation, and sense of purpose [3, 22, 23]. These age-sensitive approaches would help us better understand the potential of successful aging among those individuals who have already had success in longevity.

## Figures and Tables

**Table 1:** Population characteristics.

Characteristic	Frequency %
Age	(*N* = 1283)
90–91	44.5
92–93	25.5
94+	30.0
Gender	(*N* = 1283)
Women	81.2
Men	18.8
Marital status	(*N* = 1267, missing 16)
Unmarried	87.9
Married	12.1
Education	(*N* = 1234, missing 49)
Low	56.4
Middle	9.9
High	22.7
Higher	11.0
Place of living	(*N* = 1278, missing 5)
Community	62.5
Institution	37.5

**Table 2:** Frequency (%) of the variables composing three components of successful aging in men and women.

Variables	Men (*N* = 226–238) %	Women (*N* = 1006–1038) %	*P* value	All (*N* = 1227–1283) %
Physical component				
No heart problem	42.4	47.1	0.192	46.2
No stroke	96.2	94.4	0.250	94.7
No circulatory problems in brain	78.6	79.3	0.798	79.2
No diabetes	85.7	88.8	0.178	88.2
No arthritis	69.7	54.3	<0.001	57.2
No Parkinson's disease	99.6	98.3	0.148	98.6
No hip fracture	89.2	81.3	0.003	82.8
No dementia	66.4	59.2	0.033	60.6
No disease	14.7	10.2	0.045	11.0
2 or less diseases with no dementia	39.5	29.8	0.004	31.6
Able to see	72.8	59.9	<0.001	62.3
Able to hear	68.1	71.5	0.299	70.9
Able to see and hear	53.2	48.2	0.170	49.2
Independent in five activities	58.3	34.9	<0.001	39.2
Independent in 3 easier activities	83.0	72.5	<0.001	74.5
Criterion 1*	7.7	4.8	<0.001	5.3
Criterion 2*	28.3	26.1	0.500	26.5
Criterion 3*	24.8	19.1	0.050	20.2
Criterion 4*	33.9	23.2	<0.001	25.2
Psychological				
No depressiveness	87.8	79.6	0.004	81.2
Self-rated health average or good	72.3	61.4	0.002	63.4
Willing to live up to 100 years	42.4	24.8	<0.001	28.1
Psychological component	34.0	16.7	<0.001	20.0
Social engagement				
Met children during past 2 weeks	92.7	93.5	0.644	93.4
Phone contacts	84.6	79.4	0.070	80.4
Social component	78.8	74.4	0.166	75.2

*Criterions. Criterion 1: No disease, and able to hear and read, and independent in all five activities. Criterion 2: Less than 3 diseases, no dementia, able to hear and read, and independent in three easier activities. Criterion 3: No dementia, able to hear and read, and independent in 5 activities. Criterion 4: Able to hear and read, independent in 5 activities.

**Table 3:** Prevalence of successful aging (%) according to the six models in different socioeconomic categories.

	Models of successful aging*					
	1	2	3	4	5	6
Total prevalence	1.6	6.3	5.7	6.8	6.3	18.3
Age						
90–91	1.9	7.9	6.5	7.5	7.5	23.8
92–93	2.5	6.8	7.1	8.6	7.7	21.2
94+	0.5	3.4	3.2	4.2	3.4	7.8
*P* value	0.104	0.021	0.042	0.048	0.022	<0.001
Gender						
Men	4.7	12.4	11.1	13.2	12.4	22.3
Women	1	5	4.5	5.5	5.1	17.5
*P* value	<0.001	<0.001	<0.001	<0.001	<0.001	0.095
Marital status						
Married	3.3	11.8	11.8	14.5	12.5	24.2
Not married	1.4	5.6	4.9	5.9	5.6	17.6
*P* value	0.095	0.003	0.001	<0.001	0.001	0.03
Place of living						
Community	2.3	8.4	7.6	9.1	8.2	25.9
Institution	0.6	2.9	2.5	3.1	2.5	6.1
*P* value	0.026	<0.001	<0.001	<0.001	<0.001	<0.001
Education						
Low	1.3	4.7	4.6	6	5.8	14.4
Middle	1.6	7.4	4.9	4.9	5.7	20.5
High	2.1	7.5	7.1	8.2	7.2	22.6
Higher	3	12.5	9.6	11.9	9.6	29.1
*P* value	0.51	0.005	0.093	0.058	0.377	<0.001

*Models of successful aging. Model 1. Health and functioning criterion 1 + psychological + social. Model 2. Health and functioning criterion 2 + psychological + social. Model 3. Health and functioning criterion 3 + psychological + social. Model 4. Health and functioning criterion 4 + psychological + social. Model 5. Health and functioning criterion 3 + psychological. Model 6. Health and functioning criterion 3 + social.

**Table 4:** Associations of successful aging, according to the six models, with socioeconomic characteristics. A multivariate logistic regression model, all the predictors included in the model simultaneously. Odds ratios (OR) and 95% confidence intervals (CI).

	Models of successful aging					
	1	2	3	4	5	6
Age						
90–91	2.74 (0.59–12.76)	1.93 (0.99–3.78)	1.68 (0.83–3.4)	1.43 (0.76–2.66)	1.82 (0.93–3.57)	2.85 (1.81–4.49)
92–93	3.93 (0.82–18.89)	2.15 (1.02–4.53)	1.76 (0.91–3.42)	1.93 (1.0–3.73)	2.14 (1.05–4.40)	2.90 (1.79–4.73)
94+	1	1	1	1	1	1
Gender						
Men	1	1	1	1	1	1
Women	0.20 (0.07–0.54)	0.46 (0.26–0.82)	0.53 (0.29–0.96)	0.54 (0.31–0.94)	0.47 (0.27–0.83)	0.98 (0.06–1.50)
Marital status						
Unmarried	1	1	1	1	1	1
Married	0.85 (0.26–2.73)	1.17 (0.61–2.26)	1.60 (0.82–3.12)	1.71 (0.92– 3.16)	1.42 (0.74–2.7)	1.11 (0.69–1.81)
Education						
Low	1	1	1	1	1	1
Middle	1.64 (0.34–7.88)	1.77 (0.81–3.85)	1.16 (0.47–2.87)	0.89 (0.37–2.18)	1.10 (0.47–2.54)	1.57 (0.94–2.63)
High	1.14 (0.39–3.33)	1.28 (0.72–2.3)	1.23 (0.68–2.22)	1.10 (0.64–1.91)	0.95 (0.54–1.69)	1.45 (1.00–2.11)
Higher	1.35 (0.38–4.6)	2.03 (1.06–3.89)	1.48 (0.73–2.99)	1.45 (0.76–2.76)	1.14 (0.57–2.28)	2.00 (1.26–3.17)
Place of living						
Community	3.11 (0.89–10.8)	2.48 (1.36–4.53)	2.66 (1.39–5.05)	2.64 (1.48–4.72)	3.18 (1.68–6.0)	4.30 (2.83–6.53)
Institution	1	1	1	1	1	1
